# Biosensor-Based Detection of Calprotectin and Lactoferrin as Neutrophil-Derived Markers of Inflammatory Bowel Diseases: From Molecular Pathophysiology to Point-of-Care Platforms

**DOI:** 10.3390/ijms27062692

**Published:** 2026-03-16

**Authors:** Nikita Sitkov, Andrey Ryabko, Sergei Ivanov, Yuri Cheburkin, Alexey Kolobov, Diana Khasanova, Vladimir Nikolaev, Dmitrii Kaplun, Kamil Gareev

**Affiliations:** 1 Department of Micro and Nanoelectronics, Saint Petersburg Electrotechnical University “LETI”, 197022 Saint Petersburg, Russia; d.i.a.n.a.2109@mail.ru; 2 Institute of Power Electronics and Photonics, Saint Petersburg Electrotechnical University “LETI”, 197022 Saint Petersburg, Russia; yucheburkin@gmail.com (Y.C.); alexey.kolobov.spb@gmail.com (A.K.); 3 Almazov National Medical Research Centre, 197341 Saint Petersburg, Russia; ivanov.sv@mail.ru; 4 Ioffe Institute, 194021 Saint Petersburg, Russia; a.a.ryabko93@yandex.ru (A.R.); nkvlad@inbox.ru (V.N.); 5 Saint Petersburg State Pediatric Medical University, 194100 Saint Petersburg, Russia; 6 Institute of Human Hygiene, Occupational Pathology and Ecology, 188663 Saint Petersburg, Russia; 7 School of Computer Science and Technology/School of Artificial Intelligence, China University of Mining and Technology, Xuzhou 221116, China; dikaplun@etu.ru; 8 Department of Automation and Control Processes, Saint Petersburg Electrotechnical University “LETI”, 197022 Saint Petersburg, Russia

**Keywords:** inflammatory bowel diseases, calprotectin, lactoferrin, biosensors, nanomaterials, point-of-care testing, antibodies, aptamers, peptides, label-free detection

## Abstract

Inflammatory bowel diseases (IBD) are chronic, relapsing, immune-mediated disorders that require regular and preferably noninvasive monitoring of inflammatory activity. Fecal biomarkers of neutrophilic inflammation, namely calprotectin and lactoferrin, therefore represent key analytical targets for diagnosis and longitudinal disease management. Despite their widespread clinical use, existing publications predominantly address either their clinical relevance or individual technical solutions, without establishing a comprehensive engineering-translational framework for their biosensor-based implementation. This review bridges this gap by providing an integrative analysis of the molecular and biological nature of calprotectin and lactoferrin, the mechanisms underlying their appearance in fecal matrices, and the analytical constraints that directly influence the design of hybrid point-of-care (PoC) biosensor systems. We systematically compare major biosensing platforms, emphasizing sensor architecture, signal transduction mechanisms, and sample preparation strategies as critical determinants of sensitivity, selectivity, reproducibility, and clinical relevance. The novelty of this review lies in combining the pathophysiological context of neutrophilic inflammation with physicochemical and technological aspects of biosensor development, enabling a transition from laboratory prototypes to evaluation of real translational readiness. The practical significance resides in establishing a methodological basis for rational design of next-generation hybrid-integrated biosensor systems and outlining perspectives for digital analytics and artificial intelligence in clinically interpretable IBD monitoring.

## 1. Introduction

Inflammatory bowel diseases (IBD) are chronic immune-mediated inflammatory disorders of the gastrointestinal tract, primarily ulcerative colitis (UC) and Crohn’s disease (CD), characterized by a relapsing course, systemic manifestations, and a risk of complications that may ultimately lead to disability. UC typically involves the colonic mucosa, whereas CD is characterized by transmural inflammation and may affect different segments of the gastrointestinal tract [1]. Clinically, IBD requires not only initial diagnosis but also regular assessment of inflammatory activity, monitoring of therapeutic response, and early detection of exacerbations. Over recent decades, the epidemiology of IBD has changed substantially: the disease has acquired a distinctly global dimension, and the overall burden of IBD continues to rise in many regions of the world [2]. This trend implies a growing number of patients requiring long-term follow-up, accessible diagnostic testing, and scalable monitoring technologies. A review by the European Crohn’s and Colitis Organisation (ECCO) emphasizes that the epidemiology of IBD has undergone significant changes and is accompanied by an increasing global disease burden [3].

Because IBD in most cases requires lifelong continuous therapy, ongoing monitoring of the disease course is essential, with primary emphasis on the assessment of intestinal inflammatory activity. Such monitoring is necessary to ensure the effectiveness of treatment strategies and the achievement of therapeutic targets. Its principal role is to maintain patient well-being and to secure the resolution of mucosal inflammation, since persistent mucosal inflammation is what ultimately leads to disease-related complications [4]. Contemporary IBD management is increasingly aligned with the treat-to-target (T2T) paradigm, in which the key objective is the achievement and maintenance of efficient measures of inflammatory control rather than symptom relief alone. The STRIDE-II concept formalizes therapeutic targets and highlights that normalization of laboratory markers, including serum inflammatory markers and fecal biomarkers, constitutes a short- to intermediate-term goal of disease control [5,6].

At the same time, endoscopy and histological assessment remain the reference methods for evaluating inflammatory activity and mucosal healing, but they are invasive, costly, and poorly suited to frequent monitoring in routine clinical practice. Consequently, there remains a strong interest in noninvasive biomarkers capable of reflecting intestinal inflammatory activity and supporting decision-making both during differential diagnosis (for example, distinguishing inflammatory disease from functional disorders) and during long-term monitoring of remission and early relapse. Therefore, many studies examine various molecular markers of IBD to obtain more complete information about the patient’s condition [7,8,9,10]. Noninvasive or minimally invasive approaches are particularly attractive because they reduce the number of hospital visits. Within this framework, the most firmly established markers in clinical practice are fecal biomarkers of neutrophilic inflammation, primarily calprotectin (FC) and lactoferrin (LF) [11,12].

The role of these markers in current diagnostic algorithms is clearly reflected in the American Gastroenterological Association (AGA) clinical guidelines on the use of biomarkers in UC [13]. These recommendations emphasize that, in patients with symptomatic remission, a monitoring strategy combining symptoms and biomarkers is preferred, and they provide a practical threshold and wording for ruling out active inflammation. Despite the high clinical demand for fecal biomarkers, conventional laboratory methods for their determination (enzyme-linked immunosorbent assay (ELISA) and related formats, as well as other laboratory-based approaches) have several limitations, especially in the context of frequent and rapid monitoring. Critical constraints include sample transport logistics, the requirement for laboratory infrastructure and qualified personnel, and turnaround time. For this reason, point-of-care (PoC) testing approaches appear promising for shortening the «laboratory pathway» and thereby enabling a rapid response to possible deterioration in the patient’s condition [14]. It should also be noted that, for fecal biomarkers, the preanalytical phase (storage conditions and sample preparation) plays a substantial role and may introduce systematic bias while reducing result comparability. For example, comparison of several test systems has shown that FC concentrations may decrease markedly when samples are stored at room temperature, and that the magnitude of this decrease depends on the assay used [15].

These limitations may potentially be overcome through the development of biosensor-based and point-of-care diagnostic systems capable of shortening time-to-result without requiring laboratory infrastructure. The possibility of integrating sample preparation, rapid detection, digital readout, and intelligent interpretation of results makes such devices highly promising for implementation within modern medical decision-support systems. Recent reviews on biosensors in IBD underscore their role as rapid, potentially noninvasive tools for monitoring key biomarkers and as a technological response to the limitations of current clinical methods [16]. Therefore, the present review does not aim to recapitulate biosensor theory in a generic way, but instead focuses on the target-specific analytical and translational challenges associated with calprotectin and lactoferrin as two neutrophil-derived markers of intestinal inflammation.

From a practical view, it is important to note that although FC is the most widely used fecal biomarker, the clinical use of other markers, including LF, remains less common, which further motivates the development of technologies capable of improving the accessibility, informativity, and speed of testing [17]. In this context, the systematization of biosensor solutions for two key markers, LF and FC, appears methodologically justified, since they address the same clinical task (non-invasive monitoring of intestinal inflammation) yet may differ in their analytical bottlenecks, including matrix effects, interference, sample preparation requirements, measurement format, and operating parameters. These factors, in turn, influence the choice of biorecognition receptors, materials, and methods for signal detection and data processing. The development of an effective self-testing system capable of quantitatively determining LF and FC in stool samples, thereby allowing patients with IBD to monitor intestinal inflammatory activity independently, could improve the quality of outpatient care. A patient with IBD would be able to assess disease activity independently when specific complaints arise, based on self-measurement of these biomarker levels. In this way, the patient could make an informed decision about whether to seek medical attention. This is particularly important for patients living at a considerable distance from medical centers.

The aim of this review is to analyze current biosensor-based strategies for detecting calprotectin and lactoferrin as two clinically relevant neutrophil-derived markers of intestinal inflammation in IBD. Unlike previous reviews, which have generally focused either on the clinical interpretation of fecal biomarkers or on individual sensing technologies, the present work examines these two targets within a single molecular–engineering framework that links protein biology, fecal matrix effects, sensing architecture, and translational constraints. Particular attention is paid to detection principles, sensing-element design, sample preparation, analytical performance, and factors that determine whether a given platform can be realistically translated into hybrid-integrated point-of-care testing systems.

## 2. Calprotectin and Lactoferrin as Biological Markers of IBD

In IBD, mucosal neutrophilic inflammation is a central component of active disease and may also persist during clinically silent or subclinical phases. For this reason, effective monitoring requires markers that reflect local intestinal inflammatory activity, change in parallel with escalation or resolution of mucosal inflammation, and can be measured in a non-invasive format. Among the available fecal biomarkers, calprotectin (FC) and lactoferrin (LF) are the most established neutrophil-derived proteins in clinical practice [18,19]. Both appear in stool as a direct consequence of neutrophil recruitment to the inflamed mucosa, followed by degranulation, cell damage, and/or cell death at the site of intestinal inflammation [20,21,22].

At the same time, FC and LF should not be regarded as analytically interchangeable targets. Although both reflect neutrophil-driven inflammation, they differ in molecular organization, intracellular localization, release pathways, and interaction with the fecal matrix. These differences are important for biosensor development because they affect epitope accessibility, surface adsorption, sample preparation requirements, and the choice of biorecognition element structure and transduction format. A direct comparison of the two markers is therefore useful not only from a biological perspective but also for understanding why biosensor architectures optimized for one target cannot always be directly transferred to the other.

In active IBD, the epithelial barrier is disrupted, and the expression of adhesion molecules, chemokines, and innate immunity mediators is upregulated. This leads to the recruitment of neutrophils from the circulation and their transendothelial migration into the mucosa [23,24]. Subsequently, neutrophils migrate toward the epithelium and into the intestinal lumen, where they execute effector mechanisms: degranulation, oxidative burst, NETosis, and lysis. The result is the accumulation of neutrophilic proteins in the intestinal lumen (and, consequently, in the stool). Among these, FC and LF prove to be analytically advantageous due to their high abundance, comparative stability within the matrix, and strong functional correlation with the neutrophilic inflammatory response. The main biologically and analytically relevant differences between FC and LF are summarized in Table 1.

These differences have direct implications for assay design. FC is a smaller cytosolic complex whose measurable form may depend on Ca^2+^ concentration, transition-metal binding, and oligomerization state, whereas LF is a larger glycoprotein released primarily from neutrophil granules and is more likely to interact with matrix components or sensor surfaces through nonspecific adsorption. As a result, biosensors developed for FC often need to account for conformational and ionic effects, while LF-oriented systems may require greater emphasis on antifouling strategies, surface blocking, and sample processing standardization.

Calprotectin is a heterodimeric protein complex of the S100 family: S100A8/S100A9 [25]. It is abundantly present in the neutrophil cytosol; therefore, even moderate infiltration or cellular damage causes a marked change in fecal FC concentration, which reaches significantly high levels during pronounced inflammatory activity. At the molecular level, S100A8/A9 is a calcium-binding protein capable of forming more stable structures in the presence of divalent cations [26]. One of the most critical functions of FC is its ability to chelate transition metal ions (Mn, Zn, etc.), a property linked to its antimicrobial activity. FC not only reflects neutrophilic inflammation but is also integral to the biology of antibacterial defense, which is frequently activated in IBD due to barrier disruption and immune cell contact with microbial components [27]. Metal-binding states and oligomerization may influence conformation, epitope accessibility for antibodies, surface adsorption, and protein behavior at the biosensor interface.

Beyond its antimicrobial role, FC functions as a damage-associated molecular pattern (DAMP): released from activated or dying innate immune cells, it can potentiate inflammatory cascades (including via interactions with innate immunity receptors), thereby sustaining cell recruitment and response amplification [28]. Therefore, from an IBD diagnostic perspective, its levels rise not only as a neutrophil response but also as part of the self-sustaining inflammatory ecosystem of the mucosa. Since FC is localized primarily in the neutrophil cytosol, the main mechanisms for its release into the extracellular environment are membrane damage and/or cell death, as well as the expulsion of contents during NETosis [29,30]. Active inflammation induces neutrophil migration across the epithelium. A portion of these cells degranulate in the tissues and lumen, while others undergo lysis. Consequently, a large quantity of cytosolic proteins, including S100A8/A9, enters the intestinal lumen and mixes with fecal matter. This rationale aligns well with the classification of FC specifically as a marker of neutrophilic inflammation, rendering it particularly informative in ulcerative colitis and Crohn’s disease phenotypes characterized by a pronounced mucosal neutrophilic component.

Clinically, the correlation of FC with inflammatory activity and endoscopic endpoints is crucial. Therefore, FC is utilized as a key non-invasive indicator of inflammatory activity and a tool for predicting remission or therapeutic response. Systematic reviews highlight that FC concentration can serve as a marker of endoscopic/histological remission (in specific cohorts and protocols) [31].

Lactoferrin is an iron-binding glycoprotein of the transferrin family, possessing distinct antimicrobial and immunomodulatory properties [32]. It is capable of binding Fe^3+^, thereby limiting iron availability to the microbiota and pathogens, as well as influencing inflammatory reactivity through multiple mechanisms (antimicrobial effects, interactions with immune cells, and modulation of barrier function). Furthermore, LF is characterized by distinct surface chemistry (glycosylation, cationic regions, interaction with lipopolysaccharides and cellular receptors), making it prone to non-specific adsorption and binding to matrix components, particles, or polymers [33]. A key distinction between LF and FC lies in their intracellular localization. Lactoferrin is packaged within neutrophil granules (classically discussed as secondary/specific granules) and is released primarily via degranulation [34,35]. This granular nature is significant for interpreting the role of fecal lactoferrin in IBD. While FC is particularly sensitive to neutrophil lysis/mass destruction (due to its cytosolic abundance), LF may become detectable during the stage of active degranulation as part of the neutrophil effector program. From a clinical perspective, this supports LF as a marker of neutrophilic inflammation that is biologically closer to active granule secretion and mucosal antibacterial defense.

**Figure 1:**
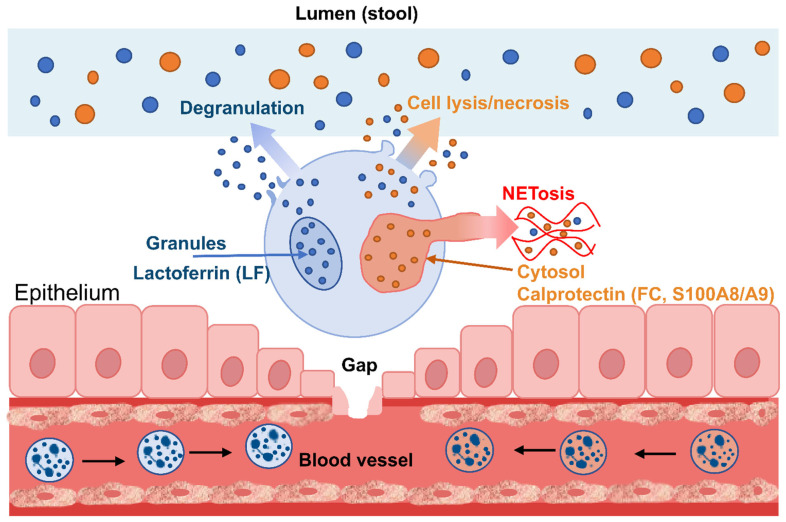
Biological scheme of the appearance of calprotectin and lactoferrin in feces in IBD.

The primary mechanisms of calprotectin and lactoferrin appearance in feces in IBD are schematically presented in Figure 1.

During mucosal inflammation, neutrophils migrate into the tissue and the intestinal lumen. Upon activation, they undergo degranulation, releasing granular proteins (including lactoferrin) into the extracellular milieu. Subsequently, as the inflamed mucosa produces exudate and the epithelium is frequently compromised, these proteins enter the intestinal lumen and mix with fecal matter. In active IBD, particularly in the presence of ulceration and/or erosions, as well as a pronounced neutrophilic response, this pathway becomes particularly intense. From a molecular perspective, LF is of interest not merely because it is released from granules, but because it performs several functions relevant to the inflamed intestine: it binds iron (thereby limiting bacterial growth), interacts with bacterial components and modulates immune cell signaling pathways [36,37,38]. Consequently, the elevation of fecal LF levels serves as a direct reflection of an activated and sustained intestinal neutrophilic response.

The fundamental reason for the value of FC and LF specifically as fecal markers lies in the spatial nature of the process. Inflammation is localized within the intestinal wall, and the products of neutrophilic activity are released directly into the lumen. This favorably distinguishes them from many serum markers of systemic inflammation, which may be associated with and amplified by concomitant extra-intestinal inflammatory processes. FC and LF levels do not merely rise during inflammation. These markers have been extensively validated against endoscopic endpoints (disease activity, mucosal healing).

The association of these markers with the pronounced neutrophilic component of inflammation positions them as the foundation for developing highly reliable non-invasive diagnostic systems for regular activity monitoring. Crucially, distinct clinical scenarios require different strategies: ruling out active inflammation, confirming a flare, monitoring therapeutic response, or preventing relapse. In each scenario, the appropriate threshold may differ, while interpretation is influenced by pre-test probability, clinical context, and test system characteristics. Consequently, researchers have increasingly focused not only on marker biology but also on analytical performance and standardization [39].

For the development of modern biosensor methods, it is critical to recognize that the fecal matrix is extremely heterogeneous (containing water, mucus, lipids, undigested food residues, etc.) and laden with potential interferents (proteases, detergents, salts, bile acids, etc.). Furthermore, marker concentrations can be influenced by pre-analytical variables, such as time-to-analysis, temperature, extraction method and buffer, homogenization protocol, and potential contamination with water or urine [40]. Samples are frequently collected at home and may remain at room temperature for hours or even days prior to extraction and analysis. Therefore, the design of modern biosensor systems must account not only for the sensing component but also for reproducible sample preparation: extraction and standardization of dilution, time/temperature control, filtration/particle removal, and the mitigation of matrix effects (e.g., via buffer composition, blocking additives, or antifouling surface coatings). An effective biosensor system is defined not only by the biochemical properties of the target markers but also by its engineering architecture, including sample preparation, microfluidics, heat and mass transfer, surface functionalization, signal readout electronics and circuitry, as well as data processing and interpretation algorithms. It is therefore logical to proceed with an examination of the fundamental operating principles of modern biosensor platforms and the strategies for their interaction with various biorecognition elements.

General principles of electrochemical, optical and other biosensor transduction have been comprehensively reviewed elsewhere [41,42,43,44,45,46]. In the present article, these platforms are discussed only insofar as they influence the analytical performance of FC- and LF-targeted assays, especially with respect to sensing-element architecture, matrix tolerance, sample preparation, and suitability for point-of-care implementation.

## 3. Biosensor-Based Detection of Calprotectin

**Figure 2:**
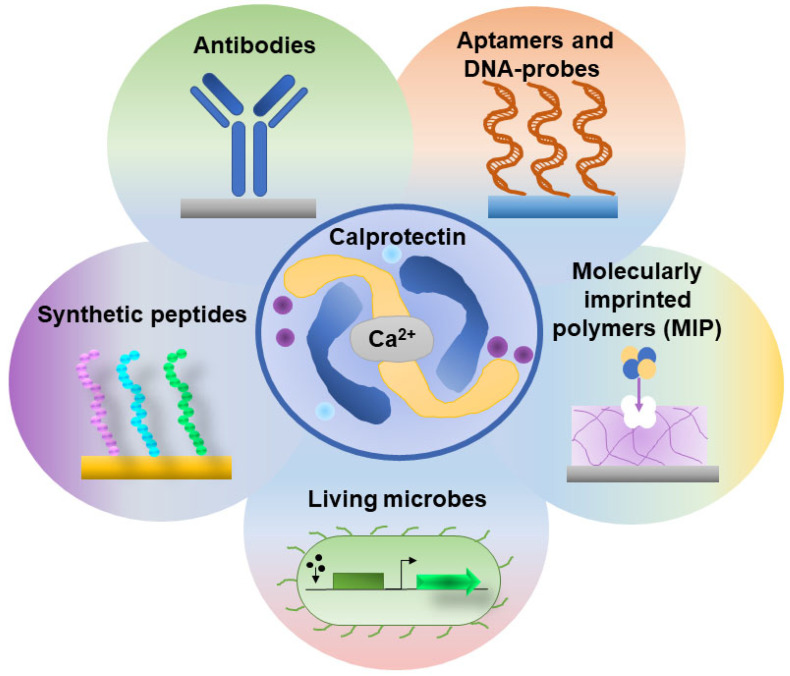
The main biorecognition elements used for the detection of calprotectin.

Fecal calprotectin represents one of the most clinically validated markers of neutrophilic intestinal inflammation. Its diagnostic value lies in the fact that active inflammation increases the influx of neutrophils into the mucosa and intestinal lumen, which in turn leads to the accumulation of FC (the S100A8/S100A9 complex) within the fecal matrix. This characteristic makes it a promising target for the development of non-invasive, rapid inflammation monitoring tools. Notably, FC exhibits Ca^2+^-dependent association and is capable of tetramerization, with its functionality being linked to metal-binding sites and transitions between oligomeric states [47]. This directly influences the specific form of the analyte present in the sample, as well as how FC interacts with surfaces (sorption) and matrix components. From the perspective of biosensor implementation for calprotectin detection, a relatively limited number of studies have been reported for this marker. Therefore, we aim to examine the key solutions to this problem, taking into account the specific properties of the target protein, covering not only fecal calprotectin but also protein detection in other biological matrices. The primary biorecognition strategies implemented for calprotectin detection are illustrated in Figure 2.

### 3.1. ELISA-Based and Related Methods

One of the key strategies for implementing calprotectin biosensing systems is the development of high-sensitivity immunoassays. An early example is the work by Damms and Bischoff [48], who presented a gold nanoparticle-based lateral flow immunoassay (LFIA). The membrane features a test line with immobilized monoclonal anti-calprotectin antibodies and a control line with anti-immunoglobulin antibodies. The sample is applied to the conjugate pad, forming an antigen-antibody complex that migrates via capillary action. At the test line, the complex is captured by secondary anti-calprotectin antibodies, while excess conjugate is fixed at the control line to validate the test. This semi-quantitative assay evaluates test line intensity on a scale of 0 to 5. At a cutoff of 50 µg/g, the rapid test demonstrated high diagnostic utility for active inflammatory bowel disease (IBD). Pre-analytical steps were detailed: 10–20 g fecal samples were collected prior to colonoscopy preparation and could be stored at 4 °C for up to 24 h. From a point-of-care (PoC) perspective, the 10–15 min readout time and lack of specialized equipment offer a significant advantage over standard ELISA, which typically requires 3–4 h.

Kido et al. [49] reported a microchip implementation of a sandwich ELISA for determining calprotectin in gingival crevicular fluid (GCF)—a matrix with extremely low volumes (0.1–1.0 µL), making conventional ELISA impractical. The authors utilized a chemiluminescent immunoassay within a microchannel: primary antibodies are fixed to the channel wall, followed by the introduction of the sample, peroxidase-labeled secondary antibodies, and a chemiluminescent substrate. A signal intensity spot is then measured using an image analyzer. The 70 × 30 mm cyclo-olefin copolymer (COC) chip features four parallel microchannels functionalized with p-nitrophenyl ester polymers for covalent antibody immobilization. This microfluidic approach reduced analysis time to approximately 35 min and sample volume to 2 µL (compared to ~100 µL for plate-based ELISA). The limit of detection (LOD) was 0.39 ng/mL. However, the authors noted that manual fluid handling and washing steps introduce signal variance, and the requirement for chemiluminescent readers limits its simplicity compared to lateral flow assay (LFA) strips.

As an alternative to antibodies, high-affinity peptide ligands are being developed. Peptide recognition elements are potentially easier to scale, easier to orient on surfaces (via self-assembled monolayers (SAM) chemistry or click chemistry), and more stable under harsh PoC conditions. Díaz-Perlas et al. [50] demonstrated synthetic peptide ligands in ELISA-like and lateral flow formats. A critical feature of this recognizer is its specificity for the tetrameric form of calprotectin; binding occurs only in the presence of Ca^2+^ and disappears for the dimer (e.g., in the presence of ethylenediaminetetraacetic acid (EDTA)). The dissociation constant (K_d_) for the linear variant was 26 ± 3 nM. X-ray crystallography revealed an extended contact area bridging two heterodimers, ensuring tetramer selectivity and potentially reducing inter-test variability common in antibody kits. In the ELISA format, the LOD was 1.6 nM (≈38 ng/mL). In the LFA variant, the peptide was used as a detection label on gold nanoparticles (streptavidin-AuNP + biotin-peptide), and in the test zone, calprotectin is captured either by an antibody (hybrid LFA) or by a second peptide (a completely “antibody-free” LFA due to the two binding sites of the tetramer). For the test strip, they obtained an LOD of 15.6 ng/mL and a working range of approximately 15.6–1000 ng/mL.

### 3.2. Electrochemical Registration of Calprotectin

Electrochemical biosensors are among the main candidates for the implementation of mass-produced PoC biosensors due to the low cost of electrode manufacturing and the possibility of miniaturization. Significantly, several recent studies report extremely low detection limits due to multi-stage signal amplification. For example, Dong et al. [51] developed an enzyme-free electrochemical sandwich immunosensor where signal amplification is achieved not by an enzyme, but by a double electrocatalytic label—a hybrid of PtNi nanoparticles immobilized on ultrathin 2D Cu-metal–organic frameworks (MOF) nanosheets Cu-TCPP(Fe) (PtNi@Cu-TCPP(Fe)). The sensor detects calprotectin by the amperometric current of H_2_O_2_ reduction (i–t) at a fixed potential of −0.4 V in acetate buffer; as the CALP concentration increases, more Ab1-CALP-Ab2-(PtNi@MOF) “sandwiches” are formed, meaning a higher catalytic rate of H_2_O_2_ reduction and a higher current. The LOD was 137.7 fg/mL. To achieve this, the measurement conditions had to be carefully selected: the pH of the working acetate buffer (optimum pH 6.8), the concentration of H_2_O_2_ (optimum 5 mM), the PtNi:Cu-TCPP(Fe) ratio (optimum 1:20), and the label incubation time (optimum 60 min). In the work by Chandio et al. [52], an electrochemical aptasensor for the determination of calprotectin is proposed, where the key contribution to sensitivity is provided by a hybrid structure consisting of high-entropy alloy nanosheets (HEANSs), additionally functionalized with amino acids (HEANSs@AAs). The authors emphasize that the multi-element composition + mesoporous architecture creates a developed surface with active centers and functional groups, which facilitates the immobilization of the NH_2_-aptamer and accelerates electron transfer, providing dual signal amplification. The claimed sensitivity range is quite wide: 5 pg/mL–100 ng/mL. The LOD was 2.02 pg/mL (S/N = 3).

Electrochemical impedance spectroscopy (EIS) is one of the most frequently used measurement techniques in biosensing. The work by Aslan et al. [53] presents a label-free impedimetric aptasensor for non-invasive diagnosis of Crohn’s disease based on the level of fecal calprotectin (S100A8/A9). An aptamer targeting S100A8 was used as a bioreceptor, ensuring specific recognition of calprotectin. Signal readout was performed using electrochemical impedance spectroscopy (EIS) by measuring the change in charge transfer resistance R_2_ (R_ct_) in a Randles-type equivalent circuit. The architecture of the developed biosensor includes layer-by-layer modification of commercial gold electrodes, on which a cysteamine layer was first formed (SAM on Au via thiol groups), then activated with glutaraldehyde, followed by immobilization of avidin, and then the biotinylated aptamer (due to the ultra-high affinity avidin-biotin interaction). Free reactive sites were blocked with HSA. Sample preparation is oriented towards real clinical practice and is relatively simple, but multi-step: stool samples are taken from patients with Crohn’s disease, then ~1 g of feces is extracted in 4 mL of extraction buffer, thoroughly vortexed, incubated at 18–28 °C for ≥5 min, then a further 1:10 dilution is made (50 µL of extract + 450 µL of buffer), vortexed again and centrifuged at 3000× *g* for 5 min, and the supernatant is collected. The LOD was 5.57 µg/g, and the LOQ was 16.89 µg/g. The main difficulty that the authors pointed out in creating the sensor is the most time-consuming part of affinity-based label-free sensors—optimizing the binding/detection time. That is why they used the chronoimpedance mode and chose 900 s as a compromise for a stable signal.

Jagannath et al. [54] demonstrated the implementation of a wearable, non-invasive biosensor for detecting calprotectin in human sweat using non-Faradaic EIS. The sensor consisted of a two-electrode system on a porous membrane patch, coupled with a portable reader. A thin film of ZnO (≈90–100 nm) was deposited onto a porous PharmChek (Fort Worth, TX, USA) patch using radio-frequency (RF) magnetron sputtering, and then silver electrodes were formed by screen printing. Biofunctionalization was performed using 3,3′-dithiobis(sulfosuccinimidyl propionate) (DTSSP) (10 mM), which was mixed with a monoclonal anti-CP antibody (50 μg/mL) and immediately applied to the electrode. Overnight incubation ensured covalent attachment (via N-hydroxysuccinimide (NHS) esters to the amino groups of the protein) and fixation to the surface (a thiol/surface binding mechanism on the ZnO interface was used). In vitro calibration of the sensor yielded a dynamic range of 0.1–10 μg/mL and an LOD of 0.1 μg/mL. The selectivity of the developed system was tested by introducing high concentrations of irrelevant inflammatory markers: CRP (C-reactive protein) (2 μg/mL), IL-6 (Interleukin-6) (1 ng/mL), IL-1β (1 ng/mL), from which the response was practically insignificant compared to calprotectin. A further development of this work was the development by Shahub et al. [55] of a non-invasive wearable two-channel electrochemical biosensor for continuous monitoring of inflammation using calprotectin and IL-6 in passive sweat. Detection was performed using non-Faradaic EIS. Sweat diffuses from the skin through a porous membrane to the sensor surface, where the target proteins bind to specific antibodies, leading to a change in impedance. Measurements were recorded every ~1–1.5 min and transmitted wirelessly to an application. The LOD for calprotectin was 10 ng/mL, and for IL-6—0.2 pg/mL. This work focused on demonstrating real-time longitudinal tracking of marker levels in healthy and chronically inflamed (UC) subjects over ~40 h.

In [56], a highly sensitive competitive electrochemiluminescent (ECL) aptasensor scheme is proposed for the determination of fecal calprotectin as a marker of IBD. The authors implemented signal amplification using DNA nanotags obtained by structural DNA nanotechnology methods: ECL-emitting “nanoflowers” are formed through rolling circle amplification (RCA), with the amplicon carrying aptamer motifs to Zinc protoporphyrin IX (ZnPPIX)/ Zinc tetrasulfophenylporphyrin (ZnTSPP) porphyrins (photoreactive centers/cofactors), which are periodically packed into a dense nanostructure and give an intense ECL response without the need for additional “lipid film-forming agents,” which simplifies the analysis. The authors emphasized that the strategy provides signal-on detection under mild conditions, yields a sub-ng/mL LOD, and the accuracy on clinical fecal samples is comparable to the standard test. In this case, sample preparation was designed to be oriented towards real clinical practice and made as “standardizable” as possible for feces: stool samples are collected using a CALEX^®^ Cap device (BÜHLMANN Laboratories, Schönenbuch, AG, Switzerland) (containing 5 mL of extractant), which provides simultaneous homogenization and dilution. Since the fecal matrix remains challenging (heterogeneity, viscosity, potential inhibitors/contaminants), the sample collection standardizer (CALEX) becomes a critical part of the methodology.

### 3.3. Optical Detection of Calprotectin

Optical methods are attractive because they are easily read by a smartphone camera and can be intuitive to the user (color scale). This area contains a large number of PoC solutions, including LFA readers and microfluidic colorimetric cartridges. For FC, photonic/hydrogel and molecularly imprinted polymer (MIP) approaches are of particular interest: they promise stability and simplified storage (compared to antibodies), although they often require careful calibration and are sensitive to swelling conditions and ionic strength. Resende et al. [57] proposed an optical label-free sensor for serum calprotectin based on a molecularly imprinted photonic hydrogel (MIPH) with an inverse opal structure. The detection method is based on a self-reporting change in structural color/reflection spectrum: when the target protein binds to the imprinted cavities, the hydrogel swells, the lattice period and effective refractive index change, and the reflection maximum shifts to the red region. The photonic template was obtained by self-assembly of monodisperse poly(methyl methacrylate) (PMMA) spheres (~200 nm) into an opal structure on glass. Then, a prepolymer mixture was introduced into the inter-sphere spaces, where calprotectin acted as a template; it was pre-incubated with the functional monomer acrylamide in PBS, and then the crosslinker Bis-Aam (bisacrylamide) and a photoinitiator were added. After curing, the PMMA spheres and the template (acetone/water mixture) were removed, forming an inverse opal hydrogel with “molds” for calprotectin. The sensitivity of the sensor in buffer was 0.06 ng/mL, and in serum about 0.07 ng/mL.

In the work of Si et al. [58] An original turn-off fluorescence detection scheme for calprotectin is proposed, based on the inhibition of the activity of the Zn(II)-dependent DNA enzyme (DNAzyme) 17E. In the presence of Zn^2+^, 17E cleaves a substrate labeled with 6-FAM (fluorescein) and the quencher BHQ1, leading to an increase in fluorescence. If calprotectin is added before the reaction, it chelates Zn^2+^ (the DNAzyme cofactor), thereby reducing the amount of free Zn^2+^, which inhibits substrate cleavage and causes the signal to decrease (turn-off). The method demonstrates a linear range of 10–200 nM and an LOD = 9.89 nM, which the authors specifically compare to tens of nM as a typical threshold for fecal CP to differentiate between IBD and functional gastrointestinal disorders. The difficulties of this method include the need for fine optimization of conditions (pH, time, Zn^2+^ concentration) and the vulnerability of the concept to any components that also bind Zn^2+^ or change the ionic strength/pH (which is critical for transitioning to real fecal samples).

The article by Chia et al. [59] demonstrates a CRISPR/Cas12a-mediated PoC assay for detecting calprotectin in mucosal samples. The presence of calprotectin increases the output signal of the CRISPR/Cas12a system in the presence of trigger-ssDNA, allowing for a quantitative correlation between protein concentration and fluorescence intensity/color signal on the strip. The authors implement two versions: a fluorescent one (requiring a reader/tablet) and a colorimetric one for POCT, where the readout is performed via a lateral flow strip. Both versions claim an LOD = 1 ng/mL and a range of 1 ng/mL–10 μg/mL. Specificity within the model is demonstrated through a panel of interferents (Interferon gamma IFN-γ, IL-6, IL-10, tumor necrosis factor TNF-α, IL-1β at 10 μg/mL and bovine serum albumin (BSA) at 10 mg/mL): the background signal for all interferents is significantly lower, while the signal for CP is approximately 45% higher and statistically distinguishable.

Lan et al. [60] propose an interesting protein engineering platform, FESCA (Fab-Enabled Split Luciferase Calprotectin Assay), for the quantitative determination of calprotectin (S100A8/A9 complex) using split-NanoLuc bioluminescent complementation (NanoBiT: SmBiT/LgBiT). Two highly specific Fab binders to calprotectin (in the key variant—the CP16-SmBiT and CP16-LgBiT pair) bind the target and thereby physically bring the luciferase fragments closer together, leading to the restoration of NanoLuc activity and a sharp increase in signal after the addition of the substrate (furimazine). Under optimized buffer conditions, the authors demonstrate a pronounced “signal-on” response and indicate that a statistically significant increase in luminescence begins at 0.1 ng/µL, while a linear range is maintained from approximately 0.1–3 ng/µL. Particular emphasis is placed on translational applicability—the same bioreceptor “core module” can be adapted to various readout form factors: from plate readers to camera/smartphone readout, as well as paper and strip formats. However, the authors show in detail that in the direct analysis of fecal extract, the signal in the solution format is sharply suppressed (up to tens or hundreds of times compared to the buffer), and this suppression is not due to “quenching” of the full NanoLuc, but rather to matrix effects on the NanoBiT complementation/reporter part. This becomes a key engineering limitation and an argument in favor of immobilized or more carefully standardized sample preparation procedures. As a practical solution, the authors demonstrate the applicability of the paper-assay and lateral-flow approach, but note the variability in the academic fabrication of paper substrates, which emphasizes the need for technological standardization when transitioning to a real PoC product.

### 3.4. Microorganism-Based Biosensors for Calprotectin

As an unconventional example of biosensor detection of fecal calprotectin, the work of Xia et al. [61] deserves attention, where the authors implemented a whole-cell biosensor based on the probiotic strain *E. coli Nissle 1917* (EcN) for monitoring inflammatory bowel disease (IBD) activity. Conceptually, this differs from immunosensor platforms, as instead of traditional biorecognition elements, it uses the bacterium’s inherent cellular response to calprotectin, which acts as a powerful metal chelator (particularly Zn/Mn) at the site of inflammation, creating “metal starvation” conditions for microorganisms and activating corresponding regulatory cascades. The authors identified transcriptional markers of this response (including the *ykgMO* promoter module) and then optimized the sensor construct to reduce background expression, obtaining the *ykgMO-IGS* variant suitable for detection in a complex matrix. Two reporter formats were used as output signals: fluorescent for reading on a plate reader/flow cytometry and bioluminescent for visualization in in vivo experiments. For the analysis of human stool, the authors used a simple co-culture approach without classical analyte extraction: the sample was homogenized in PBS to a standardized suspension and incubated with a culture of the sensor EcN in minimal medium, after which the reporter signal was recorded. The dose-response characteristic for the *ykgMO-IGS* construct showed a lower limit of detection of approximately 25 µg/g of calprotectin, and early signs of induction could be detected within about an hour, although in the routine format for clinical samples, the authors used a longer incubation time. In clinical samples, the sensor distinguished between active IBD and remission/control groups and demonstrated agreement with laboratory values of fecal calprotectin. However, this approach also has obvious limitations for a classic PoC: the need to work with a live culture, the dependence of the response on environmental conditions/metal availability, and the incubation time, which in the current implementation can be significantly longer than the minute range characteristic of express tests.

Another example of a live microbial biosensor based on the probiotic strain *Escherichia coli Nissle 1917* is described in the work of Zhu et al. [62]. Calprotectin, released during neutrophil infiltration, sequesters Zn^2+^ in the intestinal lumen. When Zn^2+^ bioavailability decreases, the repression of the transcriptional factor Zur is removed, and the Zur-controlled *Pykg* promoter is activated, triggering the output signal (superfolder green fluorescent protein, sfGFP) or a therapeutic module. The authors separately show that direct incubation of the sensor with fecal/colon suspensions in vitro often does not activate the system because there is enough free Zn^2+^ remaining in the feces to suppress *Pykg*, while activation is observed after the addition of a chelator (TPEN). This is a key practical “pitfall” and at the same time an explanation of why their system works better in vivo in areas of active inflammation, where Zn^2+^ is actually associated with calprotectin. However, since the sensor measures not calprotectin itself, but Zn bioavailability, off-target conditions are possible that also cause Zn^2+^ deficiency (diet, microbiota, hormonal/genetic factors)—the authors explicitly note the need for further verification of such scenarios.

The implementation examples of primary methods for the quantitative determination of calprotectin are summarized in Table 2.

Taken together, the reviewed calprotectin biosensors demonstrate a wide spread of analytical performance, with reported limits of detection ranging from the low fg/mL–pg/mL level in highly amplified electrochemical and luminescent systems to ng/mL or even µg/mL levels in simpler strip-based, label-free, and whole-cell implementations. Their dynamic ranges are similarly heterogeneous and may extend from relatively narrow clinically focused intervals to several orders of magnitude, depending on the biorecognition element, signal amplification strategy, and assay format. However, these numerical characteristics should not be interpreted separately from matrix tolerance. In the case of fecal calprotectin, matrix tolerance is determined by the ability of the assay to maintain stable analytical behavior in the presence of stool particulates, mucins, lipids, salts, variable extraction conditions, and matrix-dependent effects on protein conformation, oligomerization, and epitope accessibility. Therefore, for calprotectin biosensors intended for personalized point-of-care use, matrix robustness is as important as nominal sensitivity and should be considered a core performance parameter alongside LOD and dynamic range.

## 4. Biosensor-Based Detection of Lactoferrin

LF has been considered as an important biomarker of various pathological conditions for several decades, and, therefore, a number of effective methods for its quantitative determination have been proposed and tested to date [63,64]: ELISA [65], fluorescent assay [66], electrochemical assay [67,68,69], and surface plasmon resonance (SPR) assay [70]. Next, we will consider in more detail the operating principle of each of the main methods of quantitative detection of LF using examples.

### 4.1. ELISA-Based LF-Biosensors

The reference method for determining LF is based on the use of ELISA, which is one of the most well-established and still used methods for determining LF; nevertheless, it is a lengthy, multi-stage analytical procedure. The ELISA method remains one of the most established approaches for LF determination. It is sensitive and accurate, but at the same time represents a relatively lengthy multistep analytical procedure [65]. The sensitivity of the method is no worse than 0.001 μg/mL, the accuracy is close to 100%, and the reproducibility is characterized by variations of about 10% [65]. Modern cost-effective variations of the LF detection method, similar to ELISA, ensure the detection of this protein in urine at a level of no worse than 7 ng/mL with a reproducibility of no worse than 92%, a dynamic (linear) range of about 8 ng/mL–20 g/mL, and an accuracy of a few percent with an analysis time of about 3 h [71]. Li et al. [72] developed a visualized microarray method for the simultaneous, high-throughput quantitative immunodetection of several whey proteins, including LF, in samples from various milk sources. The developed method enabled the analysis of whey protein content in various raw milk samples and ultra heat treated (UHT) milk samples, including skim milk and high-calcium milk, without the need for sample preparation, including pre-enrichment or purification steps, “extraction” of target analytes from a complex matrix, and signal measurement in a “clean” medium, with good agreement with high-performance liquid chromatography (HPLC) results. A method for increasing the sensitivity of LF detection in milk is proposed using flower-shaped gold nanoparticles labeled with purified monoclonal antibodies to LF to produce test strips, which were subsequently tested on several cow’s milk samples [73].

### 4.2. Fluorescence-Based and Colorimetric LF-Biosensors

A fluorescent method for detecting lactoferrin is also well developed and can be applied, for example, to the analysis of tear fluid [74,75]. An approach for colorimetric detection of the fluorescence of a lactoferrin-terbium complex with a detection threshold of approximately 0.5 mg/mL is proposed, which can be used to analyze biomolecules present in tears by integrating a suitable probe in the detection region. Using an optical biosensor, the binding of sCD14 to LF was investigated and the potential protective effect of LF in septic shock was analyzed [76]. LF and other tear fluid components can be analyzed using a surface-enhanced Raman scattering biosensor with a design including a glass substrate and a layer of gold nanoparticles [77]. Chen et al. [66] demonstrated the feasibility of creating a highly sensitive fluorescent polarization aptasensor based on signal amplification in the presence of silver nanoparticles. This amplification strategy utilized dual recognition of two cleaved aptamer moieties and the effect of silver nanoparticles on fluorescein isothiocyanate. The achieved detection limit for LF was 1.56 pM, which is one of the best results described in the literature. Samples of biosensors based on photonic crystals integrated into microfluidic channels compatible with the 384-well microplate format were obtained and allowing kinetic analysis of protein-protein interactions (LF-heparin) in small volumes through five parallel channels (analyte flows) [67]. Gao et al. [78] determined LF concentration in tear fluid by measuring the fluorescence of inverse opal carbon with a diameter of 288 nm; linear relationship was observed between fluorescence intensity and LF concentration from 0.1 to 5 mg/mL, which corresponds to the normal range of LF concentrations in tear fluid (0.63–2.9 mg/mL).

A prototype biosensor based on fluorescence detection was developed by applying TbCl_3_ as a sensing and fluorescence-transmitting component, and NaHCO_3_ as a signal amplifier, to filter paper modified with a microfluidic pattern using an inkjet printer [79,80]. The resulting prototype provides a detection limit of 0.1–0.3 mg/mL [79,80] and enables the detection of abnormal LF concentrations in human tears. In the work [81], the authors showed that Tb^3+^ complexes with transferrin and LF act as luminescent devices for measuring pH in aqueous solutions and on polystyrene beads. A simple proof-of-concept study for the colorimetric detection of LF based on intensity and distance readings is described using paper-based assays based on the LF-induced ligand exchange reaction between a Fe^3+^-binding colorimetric indicator and a metal-binding protein [82]. A label-free aptasensor for the detection of LF in dry milk is proposed, based on the dual function of aptamers as a specific target recognition element and a fluorescent signal reporter integrated with structure-selective dyes [83]. In [84], aptamers labeled with 6-carboxyfluorescein were used as specific recognition elements and fluorescent reporter probes, LF as target proteins, and their amino acids as quenching acceptors. In the presence of LF, the aptamer was specifically configured to recognize LF by self-assembling and folding into a three-dimensional spatial structure. Lu et al. [85] first proposed an unmodified gold nanoparticle-based aptasensing assay based on pH adjustment for protein detection that can be applied universally to both basic and acidic proteins. A ratiomeric electrochemiluminescence resonance energy transfer aptasensor was proposed, which provided a new assay protocol for the detection of LF, which has the advantages of high sensitivity, low background signal and a wide analyte range [86]. Nangare et al. [87] showed that a conjugate of graphene quantum dots and molybdenum dioxide nanosheets mediated by a fluorescent nanoprobe provides an on-off fluorescence detection mechanism as well as high sensitivity and selectivity in the presence of LF. A portable platform for LF determination has been developed based on the complexation reaction between LF and Tb^3+^ [88]; fluorescence is observed after the complexation reaction is initiated, as the resulting LF-Tb^3+^ complex causes an electron in the ligand to transition to an excited state, followed by the emission of a photon in the green spectrum. This is automatically recorded by a smartphone, enabling quantitative determination of LF concentration. A prototype fluorescent sensor for Fe^3+^ based on carboxyl-rich carbon dots was developed [89]. It demonstrated satisfactory results when used as a sensor for LF, with a linear detection range of up to 6.62 μg/mL and a detection limit of 0.776 μg/mL. Wang et al. [90] used peanut protein isolates and cysteamine as precursors for the hydrothermal preparation of N, S-doped carbon dots to enhance their sensitivity and selectivity as fluorescent probes for Fe^2+^/Fe^3+^ and LF. Thus, optical methods for detecting lactoferrin have already formed a practically important direction for the analysis of tear fluid in the diagnosis of various diseases [91], since they combine sufficient sensitivity with the possibility of portable and potentially point-of-care reading.

### 4.3. Electrochemical LF-Biosensors

The study [92] demonstrates the feasibility of creating a miniature LF biosensor based on the amperometric detection principle using screen-printed carbon electrodes. The developed prototype detects LF isolated from bovine colostrum samples with a detection limit of approximately 1 μg/mL and the linear range of about 1–100 μg/mL. A design of an electrochemical biosensor is proposed based on specific non-covalent interactions between LF and metal ions chelated in a monolayer of amine-terminated terpyridine immobilized on the inner walls of track nanopores with their subsequent treatment with an iron(II) salt solution to form complexes that act as recognition elements for capturing LF molecules [93]. Campanella et al. [68] developed a prototype of biosensor for measuring lactoferrin concentration both in breast milk and in the search for commercial pharmaceutical products; in the given work, three different types of electrochemical converters were used: an amperometric electrode for determining H_2_O_2_, a gas diffusion amperometric electrode for determining O_2_ (Clark type electrode) and an ion-selective electrode for iodide. The results of LF determination using the developed biosensor [69] show that the detection limit is approximately 35 nM, and the linear range is approximately two and a half decades. BioMEMS-based systems also integrate quite well with electrochemical biosensors [94]. Debuisson et al. used silicon and polymer microfabrication to create a bioMEMS-based LF sensor designed for dielectric spectroscopic measurements on biological cells and addressing the limitations imposed by cell manipulation (shear stress effects) and high-frequency measurements (dielectric losses) [95].

A simple electrochemical method for the determination of LF is proposed, based on the use of a thin layer of MOP/3-sulfanylpropan-1-ol on the gold surface using electrochemical spectroscopy [96]. It has been experimentally demonstrated that a sensitive technique such as electrochemical impedance spectroscopy can overcome the limitations of adsorption-based immobilization methods and achieve clinically relevant threshold values for determining the concentration of protein biomarkers, including LF [97]. Huang et al. [98] developed an electrochemical immunosensor for the detection of LF by covalently attaching an anti-LF antibody to an Au electrode. A sensor chip was manufactured for studying dry eye syndrome and diabetes in a clinic setting with an experimentally determined detection range of LF from 0.1 to 10 mg/mL [99]. An electrochemical biosensor platform has been developed that enables multiplexed detection of bacteria-specific nucleic acids and host immune response proteins; its validity has been demonstrated in clinical samples [100].

Naseri et al. proposed a novel multivalent aptamer and developed the first label-free electrochemical aptasensor for the detection of human LF in urine samples with an extended linear detection range of 10–1300 ng/mL in buffer solution and high sensitivity [101]. A pilot multiplex detection of urinary pathogens and LF in clinical samples was demonstrated through the simultaneous analysis of nucleic acids (e.g., 16S rRNA) and proteins on a single sensor array [102]. Paziewska-Nowak et al. developed [103] a new affinity bioreceptor based on DNA oligonucleotides that binds to LF, which was confirmed experimentally, including using electrochemical impedance spectroscopy. The obtained results laid the foundation for further quantitative analysis of LF using the developed DNA-based bioreceptor. The linear response of the laboratory prototype impedance biosensor developed based on this concept was in the range of LF concentrations up to 625 nM with a detection limit of 1.25 nM [104]. A label-free electrochemical immunosensor based on a modified AgNP/Nafion glassy carbon electrode with antibodies to LF was developed for the detection of this biomarker in saliva in clinical concentration ranges [105]. The authors of [106] proposed a direct electrochemical biosensor platform for the selective recognition of LF in raw cow’s milk samples using methylene blue and LF in milk co-immobilized with an iron-doped multiwalled carbon nanotube/modified glassy carbon electrode Nafion.

The authors of the article [107], inspired by the concept of artificial antigen/antibody, i.e., molecularly imprinted polymer, attempted to create a sensor based on an electrochemical quartz microbalance with an imprinted epitope for the selective and sensitive recognition of LF through the epitope sequence of lactoferricin B (FF-25), extracted from the LF protein by digestion of bovine LF with gastric pepsin. This peptide of the N-terminal region was used in the mentioned work for molecular imprinting using the following monomers: zwitterionic monomer 2-methacryloyloxyethylphosphorylcholine, aromatic monomer benzyl methacrylate and 4-aminothiophenol as an electroactive monomer for the synthesis of a polymer matrix around the template epitope. Sun et al. [108] developed a design for an electrochemical biosensor module for direct integration into a smartphone or wearable device by using a reconfigurable bipotentiostat as part of an integrated platform that includes a sensing module housing a reconfigurable potentiostat designed for integration into a mobile device, an external sensor (disposable test strips, screen-printed electrodes, ion-selective electrodes, etc.), and the mobile device itself. Using a previously developed immunosensor for measuring LF concentration in milk and a superoxide dismutase-based electrochemical biosensor for measuring total antioxidant capacity, Tomassetti et al. [109] measured the antioxidant capacity of several LF-containing food supplements in cow, goat, and cow’s milk and tested the LF content of the same products to verify the validity of the developed methods. A piezoelectric quartz crystal microbalance (QCM) biosensor is proposed for LF detection based on the bond breaking method using a two-aptamer sandwich: aptamer I is anchored on the QCM electrode via Au-S bonds to capture LF and is coupled to aptamer II bound to a magnetic particle to improve mass detection [110]. Yoshida and Hayakawa [111] analyzed the amount of adsorbed LF on the surface of various materials, including titanium, stainless steel, zirconium and PMMA using the equilibrium analysis method in QCM measurement.

### 4.4. SPR-Based LF-Biosensors

Compared to the classic ELISA method for quantitative determination of LF, the SPR-based biosensor offers advantages in terms of analysis speed (from 15 min) and multiplexing capabilities [112]. Furthermore, the ability to reuse the plasmonic chip’s sensitive surface significantly reduces the cost of a single test compared to ELISA. Biofunctionalization of the transducer surface for detection of LF molecules enables multiplexed detection in the sensor, as a single SPR chip contains multiple channels distributing miniature light-emitting/analyzing/light-detecting units [112]. The paper [113] describes the design of a protein detection biosensor based on localized SPR using silica gold nanoshells coated with poly(N-isopropylacrylamide and methacrylic acid). Compared to previously described biosensors based on this effect, the developed biosensor demonstrates unprecedented shifts in the SPR signal (up to 50 nm) upon analyte binding.

Determination of LF concentration is part of the “gold standard” for the assessment of the quality of infant formula established by the Stakeholder Program on Infant Formula and Adult Nutrition, therefore studies using the SPR-based reference method are compared with the standard method performance requirements for lactoferrin for the main assay characteristics (calibration, detection limit, specificity, precision and recovery) [114]. Automated SPR biosensor analysis for the quantitative determination of lactoferrin in protein isolates, milk, colostrum and lactoferrin-supplemented infant formulas has been described for over 20 years [115,116]. The application of an immunoassay based on SPR detection is described for studying the phenomenon of LF denaturation under the influence of heat through the loss of conformational integrity of multiple epitope determinants of native LF recognized by an immobilized polyclonal antibody [117]. Jia et al. [118] demonstrated a novel method for selecting representative sequences from cluster analysis of a quasi-aptamer pool and used surface plasmon resonance imaging to perform label-free recognition analysis of different immune molecular sites between LF and aptamers with high throughput. The feasibility of using antibody fragments to achieve continuous protein biosensor analysis with spontaneous sensor reversibility was demonstrated through the use of specially developed fast-dissociating antibody fragments, enabling continuous detection of LF at nanomolar concentrations using a single-antibody fragment sensor [119]. In the work [109], for the quantitative determination of LF using the SPR effect, a biosensor design was used based on a glass disk coated with a 50 nm thick gold layer deposited on top of a 1.5 nm thick titanium layer and coated with a self-assembling monolayer with antibodies to LF chemically bound to it through a reaction with carbodiimide and succinimide.

### 4.5. The Biosensors Based on the Other LF-Detection Principles and LF-Based Biosensors

In the designs of modern biosensors, LF can act not only as a detectable biomarker, but also as the actual recognition element of a highly sensitive sensor for the presence of pathogenic microorganisms [120]. This may be important given the intrinsic activity of LF and its derivatives against pathogens. For example, Liu et al. proposed a simple strategy to produce recombinant LF in functional hosts for field application and demonstrated that at least three recombinant probiotic clones of two functional hosts could produce low-potency but functional LF, which significantly enhanced the antibacterial activity of LF-resistant hosts against various foodborne pathogens [121].

The paper [122] describes an approach to creating a new category of adjuvants based on the formation of complexes between two adjuvants with known stimulating effects on the immune system—LP and monophosphoryl lipid A—which demonstrated effectiveness in stimulating humoral and cellular immune responses to several types of antigens when tested in vivo. The kinetics of the binding reaction between monophosphoryl lipid A and LF (at an initial concentration of 50 μg/mL) were determined using surface plasmon resonance analysis using the BIAcore™ 1000 biosensor system. Bovine whey protein from crude acid whey was used by the authors of [123] to test an optimized magnetic separation protocol using iron oxide nanoparticles and a high-gradient rotor-stator magnetic separator. In addition to the application of the SPR method, in the work [119] a continuous probing method based on the movement of tethered particles was applied. Scleral lenses with multifunctional bioinspired high aspect ratio nanopillars made of biocompatible Parylene C and conformally coated with gold have been developed that provide sensory functions by concentrating lacrimal proteins in the coffee ring—lysozyme and LF at concentrations from 0 to 6 mg/mL in PBS—using droplet coating Raman spectroscopy [124].

Popplewell et al. [125] developed a strategy for immobilizing oligosaccharides for their oriented binding, monitoring the process in real time using dual-polarization interferometry (DPI) to determine the density and orientation of groups attached to the surface. Surfaces prepared with a decasaccharide (degree of polymerization 10, DD-10) from heparin were analyzed in detail for LF binding using DPI. The method revealed that LF has two modes of association with heparin-derived DP-10. Using classical electrophoretic mobility shift analysis, the possibility of direct aptasensing of LF in complex biological samples was investigated using a horizontal mini-gel system and a smartphone integrated with a portable blue-light transmittance meter [126]. The proposed approach can be extended to proteins other than LF using suitable aptamers, including for multiplexed protein detection.

**Figure 3:**
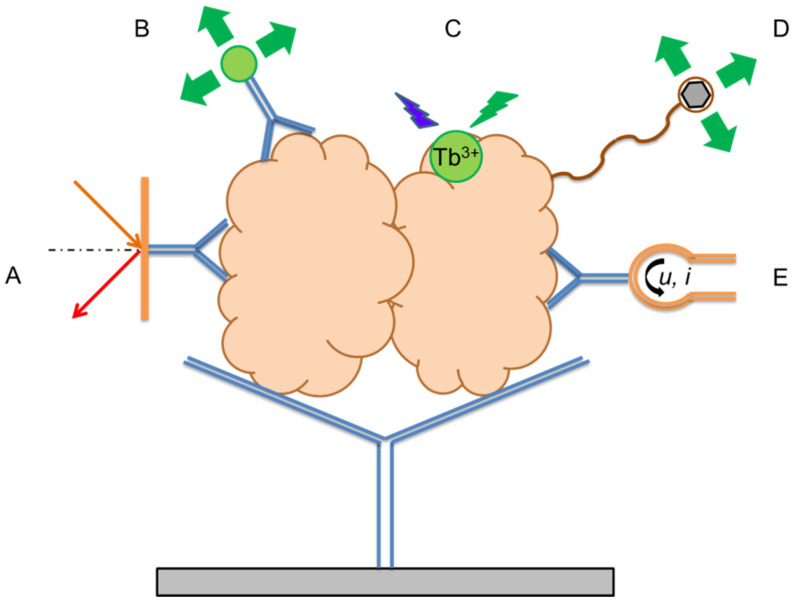
Schematic representation of the main approaches to detecting LF: (A) Surface plasmon resonance sensor; (B) Enzyme-linked immunosorbent assay; (C) Protein-Tb^3+^ fluorescence sensor; (D) DNA aptasensor with carbon dots; (E) Electrochemical (impedance) sensor.

Examples of the implementation of the basic methods for the quantitative determination of LF are given in Table 3. The physical principles on which the listed detection methods are based are schematically illustrated in Figure 3.

In the case of lactoferrin, the reported biosensor characteristics also cover a broad analytical range, from very low detection limits in amplified immunochemical and aptamer-based systems to higher but practically relevant ranges in optical, strip-based, and simplified PoC-testing formats. The achievable dynamic range varies substantially between platforms and depends on the sensing principle, surface functionalization strategy, and the extent to which the assay relies on multistep signal amplification. At the same time, the practical performance of lactoferrin biosensors is strongly influenced by matrix tolerance, since LF detection may be affected by nonspecific adsorption, interactions with complex biological components, variable extraction efficiency, and changes in interfacial behavior caused by the sample composition. As a result, the analytical performance of LF biosensors should be judged not only by their limit of detection, but also by whether they preserve reproducibility and clinically meaningful response across realistic sample conditions. In this respect, matrix tolerance and workflow robustness are critical benchmarks for assessing their translational potential.

## 5. Current Challenges and Perspectives

### 5.1. Material and Technical Barriers: Receptor Stability, Degradation, Drift, and Reproducibility

Despite the fairly intensive growth in the number of new developments in the field of biosensors for the detection of IBD markers, one of the important “bottlenecks” in their creation remains ensuring the stability of the characteristics of the biorecognition layer and the associated reproducibility of the analytical response. Since traditional immunosensors based on antibodies are prone to degradation of properties during storage [127,128], and their characteristics are also affected by the orientation and density of immobilization on the surface, which can lead to calibration drift between batches. The logistical period from manufacturing to delivery of the sensor to the analysis site can be long and does not always strictly adhere to storage conditions. This problem becomes noticeable when the system is designed for a quantitative result and must be comparable to reference clinical methods. Nevertheless, even when using alternative biorecognition agents (aptamers, peptides, MIPs, etc.), there are important limitations that developers of biosensor PoC devices need to overcome. Thus, a serious problem remains the biophysical degradation of the sensor surface, which can be expressed in oxidation or hydrolysis of functional groups, desorption of layers, rearrangement of polymers or SAMs, etc. [129,130]. In addition, biosensors may experience drift in the measurement channel, expressed in a change in the impedance baseline or the intensity of the photoexcitation source, instability of enzyme cascades, etc. These effects can be critical when several low-level changes in interfacial parameters can lead to a serious final error, loss of measurement accuracy, and impaired reproducibility. This range of problems can be compensated for using intelligent signal processing algorithms and adjusting the measurement to the sample.

### 5.2. Matrix Effects: The Complexity of the Fecal Matrix for Ensuring Analytical Functionality

For the detection of FC and LF, the fecal matrix itself presents a class of problems that are less prevalent in serum or buffer: high viscosity, the presence of lipids, mucin, food residues, microbial components, proteases, variable pH, and ionic strength. As a result, the sensor encounters fouling—rapid passivation of the surface due to non-specific adsorption of macromolecules and colloidal particles, leading to false signals and a decrease in sensitivity. Therefore, anti-fouling engineering solutions (PEG-like layers, zwitterionic coatings, hydrogels, superhydrophilic/superhydrophobic architectures, nanoporous barrier layers) become not just an improvement, but a crucial component of a functional device [131,132]. Several studies emphasize that resistance to fouling and matrix effects often determines the translational potential of a device in real-world analytical practice [133,134,135]. Along with fouling, it is also necessary to consider possible interference effects, which may manifest as competitive binding of matrix proteins to biorecognition elements or the sensor surface, distortion of the mass transfer kinetics of the analyte to the biorecognition element, and the influence of extraction buffers and detergents on the response. This is especially relevant for FC and LF, because both markers participate in a multitude of interactions (metal binding and oligomerization for calprotectin; binding to polysaccharides and surfaces, as well as resistance to proteolysis for lactoferrin), which means that the effective concentration available to the receptor on the surface may differ significantly from the nominal concentration in the homogenate.

### 5.3. Sample Preparation: Extraction, Storage, Temperature, and Transfer of Procedures to a PoC Format

Sample preparation for fecal markers is a separate technological block that is often hidden in a commercial extractor or buffer solution in laboratory test systems, but in a PoC biosensor, it becomes a full-fledged part of the device. Since pre-analytical procedures can significantly affect the measurement results, modern PoC devices should be focused on standardizing the pre-analytical stage (built-in extractor, fixed temperature, sample volume and analysis time), and also have the ability to provide algorithmic compensation for these factors. Therefore, from an engineering point of view, sample preparation in point-of-care devices should solve the following tasks: (i) reproducibly select and homogenize the sample, (ii) transfer the marker to the measurable fraction (extraction), (iii) reduce viscosity and the content of large particles (filtration/separation), (iv) reduce matrix interference (dilution and purification), (v) ensure compatibility with the transducer. It is this block that often «eats up» the advantages of the sensor: a laboratory prototype may have an excellent LOD in a buffer, but in a real sample, it loses its functionality without a well-thought-out sample preparation strategy and a reliable user scenario. It is also necessary to ensure the standardization of extraction protocols and their alignment with clinical practice. For FC tests, the problem of comparability of results between kits and the need for harmonization/reference approaches has long been discussed. Practical recommendations and comparability studies emphasize that differences in calibrators/epitopes, as well as methods and conditions of extraction, can lead to different numerical values for the same sample [5].

The duration and complexity of standard laboratory workflows may themselves affect the analytical outcome, especially for fecal biomarkers. Long protocols involving delayed extraction, multiple transfer steps, centrifugation, prolonged incubation, washing, or transport before measurement increase the risk of analyte redistribution, adsorption to plastic surfaces, matrix-dependent losses, and between-operator variability. Thus, a long protocol is not only a practical inconvenience—it may directly alter the measured result and reduce inter-assay comparability. This issue is particularly important for PoC translation, where analytical performance must be preserved under short, standardized, and user-compatible workflows.

### 5.4. Scaling Up Development: From Laboratory Prototype to Ready-to-Use Product

The existing gap between academic publications and clinical implementation is due to the fact that many developers often focus on performance indicators that are optimal from their point of view (e.g., minimum detection limit, wide dynamic range in the buffer, absence of non-specific binding for key interferents), but do not address the task of transferring their developed device/technology to the real sector. To ensure such a transition, developers need to create a technologically reproducible architecture, the manufacturing of which relies on highly standardized technological processes (e.g., micro- and nanoelectronics technologies, microfluidics, screen printing, etc.), materials and reagents with stable quality and supply chain. Standardized and consistent quality control for each batch of devices is also necessary. Platforms where high sensitivity is achieved through multilayer nanocomposites and amplification cascades are particularly complex. Each additional layer increases the variability of characteristics and reduces the yield of usable devices. Therefore, in PoC (point-of-care) architecture, engineering simplicity and reliability are increasingly valued, with a minimum number of stages, a minimum number of reagents, rapid analysis, and clear algorithms for data reading and analysis.

### 5.5. Certification, Clinical Validation, and Integration into the Diagnostic Environment

The regulatory framework for IVD devices and PoC tests has become stricter in recent years, as developers are required not only to demonstrate analytical performance but also to prove clinical utility in a given application scenario. In the European context, this is related to IVDR, which emphasizes the need for performance evaluation as a combination of scientific validity, analytical and clinical effectiveness. For PoC devices, human factors and usability issues are additionally critical: user errors during sample collection, extraction, and application, incorrect incubation time, incorrect result reading, and lighting/temperature conditions. Therefore, in real-world implementation trajectories, not only laboratory validation but also testing in home use, in outpatient settings, and in conditions of limited laboratory infrastructure are important. At the level of clinical integration, there are also requirements for accredited quality management frameworks for PoC testing (staff training, quality control, traceability of results, and responsibility of the laboratory service). National strategic guidelines on POCT explicitly emphasize the need to implement PoC in a managed and accredited framework to ensure the safety and quality of testing [136,137,138]. The interpretation of the result of such a test should be based on the clinical context and take into account the risks of false results at low pre-test probability [139,140]. Therefore, FC/LF biosensors must fit into clinical quality control and result interpretation processes. Biosensors for determining FC/LF should support clinically correct scenarios (e.g., confirmation of exacerbation vs. screening), offer clear rules for retesting, take into account variability, and, if possible, integrate with intelligent decision support systems (symptom diaries, therapy, telemedicine).

### 5.6. Integration with Artificial Intelligence and Machine Learning

The application of AI/ML in biosensing is developing in two key directions. The first area of focus is improving raw signal processing: noise suppression, drift compensation, extraction of informative features (especially in EIS and multi-frequency methods), and identification of outliers/sample preparation errors. Many reviews on ML for electrochemical sensors note that machine learning allows the conversion of complex multidimensional signals into stable and clinically describable values, and is particularly useful when working with complex matrices and in multi-sensor measurements. The second direction is the interpretation of results at the patient level: combining biomarkers with symptoms, treatment data, and patient history to predict the risk of exacerbation or response to treatment. Therefore, to ensure a personalized approach to diagnosis, it is necessary to enable the adaptation of results and the training of analytical algorithms on data for a specific patient. However, there is a serious limitation to the application of AI components, related to the risks of bias due to uneven data, as well as the risk of overfitting on “ideal” laboratory datasets, which necessitates traceability and interpretability (especially if the algorithm influences clinical decisions). Therefore, an approach where ML is used as a quality assurance (QA) module, while clinical interpretation remains conservative and transparently validated by a specialist, seems promising.

### 5.7. Architectonics and Design of Biorecognition Elements

Modern digital infrastructure is gradually shifting the development of biorecognition elements and sensor interfaces from a trial-and-error approach to rational design, where key decisions (ligand type, immobilization format, buffer, layer geometry and porosity, mass transfer conditions) are pre-tested in silico. In recent years, this direction has received a significant boost from universal models for predicting the three-dimensional structure and interactions of multicomponent systems. For example, AlphaFold 3 is positioned as an approach capable of predicting the structure of complexes involving proteins and ligands, which is fundamentally important for biosensing, where the state of the analyte is determined not only by the sequence but also by the environment (metals, pH, ionic strength) and the form of complex formation [141]. RoseTTAFold All-Atom (RFAA) and derivative diffusion models are developing along similar lines, specifically describing assemblies including proteins, nucleic acids, small molecules, metals, and covalent modifications [142]. In real PoC matrices, these details often determine the accessibility of epitopes and the stability of recognition. An important task is also modeling the interaction of the ligand with the analyte and binding scenarios (including competition, multisite binding, and the influence of conformational states). Integrative docking platforms (e.g., HADDOCK3 [143]), which allow for experimental constraints and the construction of complex ensembles, remain in demand here. A separate class of problems involves the orientation of the receptor and analyte at the sensor surface, as well as their behavior in porous/hydrogel matrices. Here, models that take into account the limitation of mobility and steric effects in the immobilized layer, charge screening and specific binding of ions/metals, protein adsorption to the matrix, and fouling play a key role. At the molecular physics level, such effects are analyzed using molecular dynamics methods and related approaches [144,145,146]. At the device level, methods for evaluating mass transfer and binding kinetics in the real geometry of the sensor layer and microfluidics can be applied [147,148]. Finally, the field of generative design (for proteins/peptides and hybrid systems) is rapidly growing, where receptors are designed for specific structural requirements: accessibility of the binding site, compatibility with immobilization, resistance to ionic strength, and the presence/absence of metals. Diffusion models of the RFdiffusion family have shown that the generative approach can create structures with specified motifs [149]. The comprehensive use of computerized approaches allows for pre-optimization of not only affinity but also tolerance to PoC conditions (buffer, pH, metals, surface attachment, packing density), as well as designing receptors that are initially oriented towards operation in a porous/hydrogel layer and in the presence of matrix interferences.

### 5.8. Formation of Multimodal Platforms

IBD monitoring rarely relies on a single marker in isolation from the clinical context. Therefore, multisensor platforms that can quickly and accurately measure FC and LF (and potentially CRP, MPO, or microbiome markers) in a single architecture are a strong prospect. Technologically, this can be implemented as a diagnostic matrix test system or a modular device with parallel sample preparation channels or disposable cartridges for different markers. The complexity of providing multimodal analysis is reflected in the increased influence of cross-interference, differences in optimal buffer and incubation conditions, and the need for calibration of individual channels and individual signal processing algorithms. Therefore, a modern PoC biosensor must possess an intelligent hardware and software core capable of optimizing sample preparation and analysis processes for a specific marker.

## 6. Conclusions

The importance of biosensor detection of molecular disease markers lies in the fact that rapid, quantitative, and clinically interpretable analysis can enable personalized monitoring of chronic conditions, promptly recognizing exacerbations and adjusting therapy. This is particularly important for ulcerative colitis and Crohn’s disease, as these conditions tend to be recurrent, and reference methods for assessing mucosal health (such as endoscopy and histology) remain invasive, costly, and poorly suited for frequent, dynamic monitoring. Under these conditions, fecal markers of neutrophilic inflammation, primarily calprotectin and lactoferrin, are becoming key molecular indicators of inflammatory activity and the basis for noninvasive monitoring of remission and exacerbations.

This review differs from most previous publications in that it examines biosensor detection of calprotectin and lactoferrin within a unified molecular, engineering, and translational framework. The analysis showed that demonstrating a low detection limit in a buffer is not sufficient for IBD monitoring systems. Reproducible detection in a complex fecal matrix, robustness to sample preparation variations, accurate interpretation of clinical thresholds, and alignment of analytical characteristics with point-of-care requirements are essential. Sensitivity is determined not only by receptor affinity but also by the architecture of the sensing layer, mass transfer, binding kinetics, the level of nonspecific adsorption, and the signal amplification strategy.

The practical value of this review lies in the formation of a methodological basis for the rational design of next-generation biosensor systems focused on the detection of neutrophil markers of intestinal inflammation. A comparison of calprotectin and lactoferrin showed that differences in their structure, stability, and interaction with the matrix require a differentiated choice of biorecognition element, detection format, and sample preparation strategy. Thus, the proposed concept, which considers the development of biosensor diagnostic systems from the pathophysiological significance of a molecular marker to an engineering-reproducible PoC solution, can be translated to other diagnostically significant analytes.

## Figures and Tables

**Table 1:** Comparative characteristics of fecal calprotectin and lactoferrin as biosensor targets in IBD.

**Parameter**	**Calprotectin (FC)**	**Lactoferrin (LF)**
Molecular nature	S100A8/S100A9 heterodimer; Ca^2+^-dependent tetramerization possible	Iron-binding glycoprotein of the transferrin family
Approximate molecular mass	~24 kDa as heterodimer; ~48 kDa as tetrameric complex	~80 kDa
Main intracellular localization in neutrophils	Cytosol	Specific granules
Main release mechanism in intestinal inflammation	Predominantly associated with neutrophil damage, lysis, NETosis, and inflammatory cell turnover; also rises with intense infiltration	More directly associated with active degranulation and granule exocytosis; may also increase with neutrophil damage
Key biochemical feature relevant to biosensor systems	Ca^2+^-dependent oligomerization; strong transition-metal binding; conformation may vary with ionic conditions	Glycosylated, cationic protein with iron-binding properties; more prone to surface and matrix interactions
Stability in fecal matter	Generally regarded as comparatively stable in stool and therefore widely standardized for routine clinical use	Clinically useful but less widely standardized; analytical behavior may be more affected by matrix interactions and handling conditions
Typical analytical role in IBD	Most widely used fecal marker of neutrophil-driven intestinal inflammation	Established complementary fecal marker of neutrophilic inflammation
Potential matrix-related challenge	Dependence on oligomeric state, ion composition, and metal binding; possible changes in epitope accessibility	Greater tendency toward nonspecific adsorption and interaction with matrix components due to protein size and surface properties

**Table 2:** Examples of the implementation of basic methods for the quantitative determination of calprotectin in various biological matrices.

**Basic Method of Detection**	**Structure and Composition of** **Sensing Element**	**Limit of Detection**	**Main Features**	**Refs.**
Lateral-flow immunoassay (LFA)	LFA strip with test line coated by anti-calprotectin monoclonal antibodies + control line with anti-immunoglobulin antibodies; conjugate pad contains dried gold-conjugated anti-calprotectin antibodies	None	Patient-friendly, fast (~10–15 min), no special equipment.	[48]
ELISA	COC microchip (70 × 30 mm) with four microchannels; surface polymer bearing p-nitrophenyl ester for covalent Ab coupling; primary anti-calprotectin Ab deposited as spots via piezoelectric inkjet printing; sealed with PMMA adhesive film.	2 ng/mL	Uses only 2 µL sample per assay; total assay time ~35 min; enables 4 samples per chip; requires chemiluminescence imaging equipment.	[49]
ELISA + LFA	High-affinity synthetic peptide ligand (linear peptide 3; tetramer-specific, K_d_ ~26 nM) obtained by phage display; in LFA–streptavidin-coated AuNP conjugated with biotin-peptide on conjugate pad; nitrocellulose membrane with test line (anti-CP Ab or neutravidin-bound biotin-peptide) and control line (calprotectin).	15.6 ng/mL (LFA)	Homogeneous, chemically synthesized ligand and improved thermal stability; requires buffer/storage optimization for AuNP conjugate stability.	[50]
Amperometric	Golden electrodes modified with multi-walled carbon nanotubes Au@MWCNTs → immobilized capture antibody Ab1 → BSA blocking → target CP → PtNi@Cu-TCPP(Fe)-Ab2 bioconjugate as catalytic label (2D Cu-TCPP(Fe) MOF nanosheets + PtNi nanospheres.	137.7 fg/mL	Dual electrocatalysis and high-conductivity base layer drive amplification; need for careful optimization (pH, H_2_O_2_, PtNi:MOF ratio, incubation time); real-sample work uses 100× serum dilution.	[51]
Electrochemical	Amino acid–functionalized high-entropy alloy nanosheets (HEANSs@AAs) providing mesoporous, multielemental active surface; immobilized NH_2_-aptamer for calprotectin capture.	2.02 pg/mL	Dual signal amplification (accelerated electron transfer + improved surface reactivity).	[52]
Label-free electrochemical impedance spectroscopy (EIS)	Gold nanoparticle electrode (DropSens 110GNP) modified layer-by-layer: cysteamine SAM → glutaraldehyde activation → avidin → biotinylated DNA aptamer (targets S100A8) → HSA blocking.	5.57 µg/g	Rapid (15 min) label-free assay; selectivity assessed vs. isolated S100A8 and S100A9.	[53]
Non-faradaic EIS	Wearable porous patch with ZnO thin film (90–100 nm, RF sputtering) + screen-printed Ag electrodes (two-electrode); immunolayer: DTSSP 10 mM + anti-CP Ab 50 μg/mL, overnight incubation.	0.1 μg/mL	Measurement directly in sweat, validated against ELISA; selectivity vs. CRP/IL-6/IL-1β.	[54]
Non-faradaic EIS	Removable sweat sensor strip + wearable reader; ZnO-coated substrate functionalized with DTSSP-linked capture antibodies (calprotectin Ab and IL-6 Ab in separate chambers); sensors lyophilized and vacuum-packaged for storage.	10 ng/mL	Continuous, noninvasive longitudinal tracking; no external sample handling; storage stability shown over 7 days at 4 °C with intra/inter-assay CV < 11%.	[55]
Electro chemiluminescence (ECL)	ECL-active DNA scaffold (ZnPDF) hosting Zn-porphyrin cofactors (ZnPPIX/ZnTSPP aptameric loci); calprotectin-binding aptamer motif (AptA8) incorporated/used in probe design; electrochemical readout in PBS/KCl with H_2_O_2_ co-reactant on a three-electrode setup.	0.419 ng/mL	Strong signal amplification via rolling circle amplification nanotags, signal-on design, high matrix viability.	[56]
Optical biosensing	Label-free optical photonic sensing via reflection peak (Bragg diffraction) red-shift caused by swelling of a molecularly imprinted photonic hydrogel (MIPH) upon binding serum calprotectin	0.06 ng/mL (PBS) and 0.07 ng/mL (serum, 1000× diluted).	Fast, label-free, reagentless readout; selectivity shown vs. CRP; sample handling simplified to dilution but requires 1000× serum dilution and 40 min incubation.	[57]
Fluorescent	Turn-off fluorescent assay via inhibition of Zn(II)-dependent 17E DNAzyme activity by calprotectin Homogeneous solution sensor: 17E DNAzyme + dual-labeled substrate in HEPES/NaCl buffer; reaction stopped by EDTA and read on fluorimeter.	9.89 nM	Enzyme-free; requires pH optimization (optimum pH 8.0) and digestion time (30 min), pre-incubation with CP+Zn^2+^ (30 min).	[58]
Fluorescent + colorimetric	Single-pot tube reaction: Cas12a protein + gRNA + quenched ssDNA reporter (Texas Red/BHQ2 for fluorescence; FAM/biotin reporter for LFA) + trigger ssDNA.	1 ng/mL	Tube format + LFA; specificity tested on a panel of cytokines/proteins (IFN-γ, IL-6, IL-10, TNF-α, IL-1β, BSA); mixture stability at 4 °C for up to 4 weeks and good reproducibility between batches.	[59]
Bioluminescent	Two anti-calprotectin Fabs (CP16 ± CP17) genetically fused to SmBiT and LgBiT; detection after furimazine addition; formats: solution + strip-based LFA (biotin-streptavidin immobilization) + paper-based cellulose assay.	0.1 ng/µL	Multi-readout (plate/CCD/smartphone); strong stool-matrix inhibition in solution (50–170× suppression); Ca^2+^-dependence; hook effect.	[60]
Whole-cell biosensor	Engineered probiotic *E. coli Nissle 1917* using calprotectin-responsive promoter (ykgMO-IGS) driving reporter output (sfGFP or *lux*CDABE). Signal readout by fluorescence/bioluminescence after coculture with stool/in vivo transit.	25 µg/g	No separate extraction step (vs. ELISA); can operate in complex stool matrix; early activation detectable ~1–3 h (flow cytometry); performance depends on media/metal availability; needs culture/readout equipment.	[61]
Living microbial biosensor	Engineered *E. coli Nissle 1917* carrying a *Zur–Pykg* genetic circuit (sfGFP output) optimized by tuning zur expression and a two-plasmid integrase-based memory switch that irreversibly flips sfGFP orientation for permanent readout; therapeutic variants replace sfGFP with YebF–IL10 (secIL10) and include asd for plasmid stability without antibiotic selection.	activation threshold ~10 µg/mL	Non-invasive in vivo sensing; “memory” enables recording patchy inflammation; sense-and-respond module (secIL10) ameliorates DSS colitis.	[62]

**Table 3:** Basic methods for the quantitative determination of LF in biological environments and examples of their modern implementation.

**Basic Method of Detection**	**Structure and Composition of** **Sensing Element**	**Limit of Detection**	**Main** **Advantage**	**Refs.**
ELISA and ELISA-mimic	Biotin conjugation with LF bound to the plastic receptor	0.001–0.010 μg/mL	High sensitivity	[71]
	Visualized microarray for quantitative immune-detection	30 ng/mL		[72]
	Immunoassay based on monoclonal antibodies and Au nanoflowers	2.4 ng/mL		[73]
	Commercial ELISA kit	–		[74]
Fluorescence and colorimetric	Polydimethylsiloxane-based contact lens with adhesive terbium-contrast	0.25–0.5 mg/mL	Cost-effectiveness	[75]
	LF-binding sCD14 in a resonant optical biosensor	10 nM		[76]
	Glass substrates with Au NPs for surface-enhanced Raman scattering	–		[77]
	Photonic crystal biosensor integrated microfluidic chip	3 μg/mL		[67]
	TbCl_3_ and NaHCO_3_ deposited onto microfluidically patterned filter paper with an inkjet printer	0.3 mg/mL		[79,80]
	Inverse opal carbon rod-based sensors attached to the eyelids	0.1 mg/mL		[78]
	Tb^3+^ immobilized on polystyrene	–		[81]
	Colorimetric Fe^3+^–indicator complex with core–shell structured poly(styrene-block-vinylpyrrolidone) NPs in a microfluidic paper-based sensor	110 μg/mL		[82]
	Self-assembly fluorescence aptasensor based on the specific embedding dye PicoGreen	3 nM		[83]
	Self-responsive 6-Carboxyfluorescein aptasensor	0.46 μg/mL		[84]
	AuNPs-based aptasensing assay	3.66 pM		[85]
	Ratiometric electrochemiluminescence resonance energy transfer aptasensor	42 fg/mL		[86]
	Graphene quantum dots and manganese dioxide nanosheets-based fluorescent nanoprobe	1.69 ng/mL		[87]
	A portable platform for LF detection based on the complexation reaction between LF and Tb^3+^	120 μg/mL		[88]
	Fluorescent sensor based on carboxyl-rich carbon dots	0.776 μg/mL		[89]
	Fluorescent sensor based on N, S-doped carbon dots	1.92 μg/mL		[90]
	Fluorescence polarization aptasensor based on bivalent aptamers and Ag NPs	1.56 pM		[66]
Electrochemical and impedance	Carbon screen-printed electrodes	1–10 μg/mL	Miniaturization	[92]
	Metallic ion chelated in an amine-terminated terpyridine monolayer inside nanopores	–		[93]
	Varios amperometric electrodes as transducers	0.035 nM		[68,69]
	Facile electrochemical LF detection based on a thin layer of MOP/3-sulfanylpropan-1-ol on the gold surface	65.2 nM		[96]
	Anti-LF IgG immobilized onto screen-printed carbon electrodes	50 μg/mL		[97]
	LF monoclonal antibody immobilized on a gold electrode	4.9 pg/mL		[98]
	Spin-coated composite of graphene nanoplatelets and amphiphilic polymer	0.1 mg/mL		[99]
	Analogous electrochemical sandwich assay based on capture and detector antibodies	2–20 pM		[100]
	Multivalent aptamer immobilized on the screen-printed gold electrode	0.9 ng/mL		[101]
	Biosensor array with alkanethiolate self-assembled monolayer	145 pg/mL		[102]
	Nonaptamer-type immobilized DNA oligonucleotide bioreceptor	1.25 nM		[103,104]
	AgNPs/Nafion–modified glassy carbon electrode with anti-LF	25 ng/mL		[105]
	Multiwalled carbon nanotube/Nafion modified glassy carbon electrode	3.2 μM		[106]
	An epitope imprinted electrochemical quartz crystal microbalance sensor	5.25 nM		[107]
	Gold electrodes functionalized with anti-human LF	1 ng/mL		[108]
	Antibodies to LF immobilized in the membrane Immobilon	70 nM		[109]
	The quartz crystal microbalance with the thiol-modified aptamer immobilized on the gold electrode surface through an Au-S bond	8.2 ng/mL		[110]
	Microfluidic system with the gold nanowire for high frequencies and nanoprobes for low frequencies	0.5 μM		[95]
SPR	Biofunctionalized nanoplasmonic grating	1 μg/mL	Label-free	[112]
	An immunoassay based on an interaction with an immobilized anti-lactoferrin antibody	0.8 mg/hg		[114]
	Goat and rabbit anti-bovine lactoferrin antibody immobilized on a sensor chip	19.9 μg/mL		[115,116]
	SPRi microarray chip with aptamer of LF	1 μg/mL		[118]
	Biofunctionalized particles tethered to a biofunctionalized substrate	10 nM		[119]
	Gold coated with a self-assembled monolayer containing chemically bonded antibodies to LF	0.28 μM		[109]
	Silica gold nanoshells coated with poly(N-isopropylacrylamide and methacrylic acid)	16 μg/mL		[113]

## Data Availability

No new data were created or analyzed in this study.

## Abbreviations

The following abbreviations are used in this manuscript:

AGA	American Gastroenterological Association
AI	Artificial Intelligence
BSA	Bovine Serum Albumin
CD	Crohn’s Disease
C_dl_	Double-layer capacitance
CL	Chemiluminescence
CV	Cyclic Voltammetry
DNA	Deoxyribonucleic Acid
DPV	Differential Pulse Voltammetry
ECL	Electrochemiluminescence
ECCO	European Crohn’s and Colitis Organisation
EIS	Electrochemical Impedance Spectroscopy
ELISA	Enzyme-Linked Immunosorbent Assay
FC	Fecal Calprotectin
FL	Fecal Lactoferrin
GI	Gastrointestinal
IBD	Inflammatory Bowel Disease
IDE	Interdigitated Electrode
LFA	Lateral Flow Assay
LF	Lactoferrin
LoD	Limit of Detection
ML	Machine Learning
MIP	Molecularly Imprinted Polymer
NETs	Neutrophil Extracellular Traps
PBS	Phosphate-Buffered Saline
PoC	Point-of-Care
QCM	Quartz Crystal Microbalance
RCA	Rolling Circle Amplification
R_ct_	Charge Transfer Resistance
RNA	Ribonucleic Acid
SAM	Self-Assembled Monolayer
SAW	Surface Acoustic Waves
SERS	Surface-Enhanced Raman Spectroscopy
SPR	Surface Plasmon Resonance
T2T	Treat-to-Target
UC	Ulcerative Colitis
